# Proxy Measures of Fitness Suggest Coastal Fish Farms Can Act as Population Sources and Not Ecological Traps for Wild Gadoid Fish

**DOI:** 10.1371/journal.pone.0015646

**Published:** 2011-01-17

**Authors:** Tim Dempster, Pablo Sanchez-Jerez, Damian Fernandez-Jover, Just Bayle-Sempere, Rune Nilsen, Pal-Arne Bjørn, Ingebrigt Uglem

**Affiliations:** 1 Department of Zoology, University of Melbourne, Parkville, Victoria, Australia; 2 SINTEF Fisheries and Aquaculture, Trondheim, Norway; 3 Department of Marine Sciences and Applied Biology, University of Alicante, Alicante, Spain; 4 NOFIMA, Tromsø, Norway; 5 Norwegian Institute for Nature Research, Trondheim, Norway; Institut Pluridisciplinaire Hubert Curien, France

## Abstract

**Background:**

Ecological traps form when artificial structures are added to natural habitats and induce mismatches between habitat preferences and fitness consequences. Their existence in terrestrial systems has been documented, yet little evidence suggests they occur in marine environments. Coastal fish farms are widespread artificial structures in coastal ecosystems and are highly attractive to wild fish.

**Methodology/Principal Findings:**

To investigate if coastal salmon farms act as ecological traps for wild Atlantic cod (*Gadus morhua*) and saithe (*Pollachius virens*), we compared proxy measures of fitness between farm-associated fish and control fish caught distant from farms in nine locations throughout coastal Norway, the largest coastal fish farming industry in the world. Farms modified wild fish diets in both quality and quantity, thereby providing farm-associated wild fish with a strong trophic subsidy. This translated to greater somatic (saithe: 1.06–1.12 times; cod: 1.06–1.11 times) and liver condition indices (saithe: 1.4–1.8 times; cod: 2.0–2.8 times) than control fish caught distant from farms. Parasite loads of farm-associated wild fish were modified from control fish, with increased external and decreased internal parasites, however the strong effect of the trophic subsidy overrode any effects of altered loads upon condition.

**Conclusions and Significance:**

Proxy measures of fitness provided no evidence that salmon farms function as ecological traps for wild fish. We suggest fish farms may act as population sources for wild fish, provided they are protected from fishing while resident at farms to allow their increased condition to manifest as greater reproductive output.

## Introduction

An ecological trap arises when an artificial habitat is introduced into a natural environment, attracts animals to its vicinity and the subsequent association leads to negative ecological consequences for the animal [1]. Animals may prefer an artificial habitat over natural habitats if it mimics the set of ecological cues which signify a good quality habitat, despite other ecological processes rendering the habitat of low quality and leading to poorer reproduction or survival. Robertson and Hutto [2] suggest that ecological traps derive from habitat alteration that operates in one of three ways; (1) increasing the attractiveness of an environment by enhancing the set of cues that animals recognise as attractive; (2) decreasing the suitability of a habitat; or (3) doing both (1) and (2) simultaneously. Alternatively, artificial habitats of high quality, where individuals increase in condition, reproduce better or have improved survival, all of which may ultimately lead to positive population growth rates, act as population sources.

Objective testing of whether ecological traps exist is well embedded in the literature concerning terrestrial systems [2], yet few studies have investigated whether they exist in marine environments. Artificial structures that aggregate fish (fish aggregation devices; FADs) have been previously suggested to act as ecological traps by acting as a super-stimulus and misleading fish to make inappropriate habitat selections [3]. Coastal sea-cage fish farms are widespread artificial structures in coastal waters, producing over 2.5 million tons of fish each year [4]. They have previously been described as analogous to FADs, attracting and aggregating large assemblages of wild fish in their immediate vicinity [5]. Attraction and aggregation of tons of wild fish to the immediate surrounds of Norway's coastal salmon farms [5], [6] meets Robertson and Hutto's [2] first condition for the formation of an ecological trap. However, whether the fish farm area is poorer in habitat quality for wild fish than natural adjacent habitats, thus meeting Robertson and Hutto's [2] second condition, remains unknown. Relative habitat quality is a key component in determining the extent to which fish farms may act as population sources or ecological traps for wild fish.

Along the Norwegian coastline, 1198 coastal sea-cage salmonid farm concessions used 1.2 million tons of fish food to produce 829 000 t in 2008 [7]. Farming is concentrated in particular fjords, with farms spaced several kilometres apart. Wild saithe are the most abundant species associated with salmon farms within fjord systems [6], [8]. Saithe use farms as a loose network of preferred habitats, moving repeatedly among farms and remaining resident at specific farms for weeks to months [9]. Atlantic cod are also attracted to fish farms in number [6] and may reside in their vicinity for months at a time [10], [11]. Attraction of wild fish to salmon farms is likely to have a range of fitness consequences due to the modified environment fish farms induce, both in the altered trophic network around farms and the close proximity of hundreds of thousands to millions of farmed salmonids. Diet, body fat content, fatty acid composition and parasite loads may all be altered when wild fish closely associate with farms [12], [13], [14]. Simultaneous analysis of this suite of factors at an extensive number of locations is required to resolve whether farms function as population sources or ecological traps [15].

Here, we tested the hypotheses that the diets, indices of condition and parasite loads of cod and saithe associated with salmon farms differed from those of fish present at locations distant from salmon farms. To ensure broad generality of the results, we sampled fish in three intensive fish farming areas along the latitudinal extent of salmon farming in Norway (59°N to 70°N).

## Materials and Methods

### Study locations and experimental design

**Figure 1:**
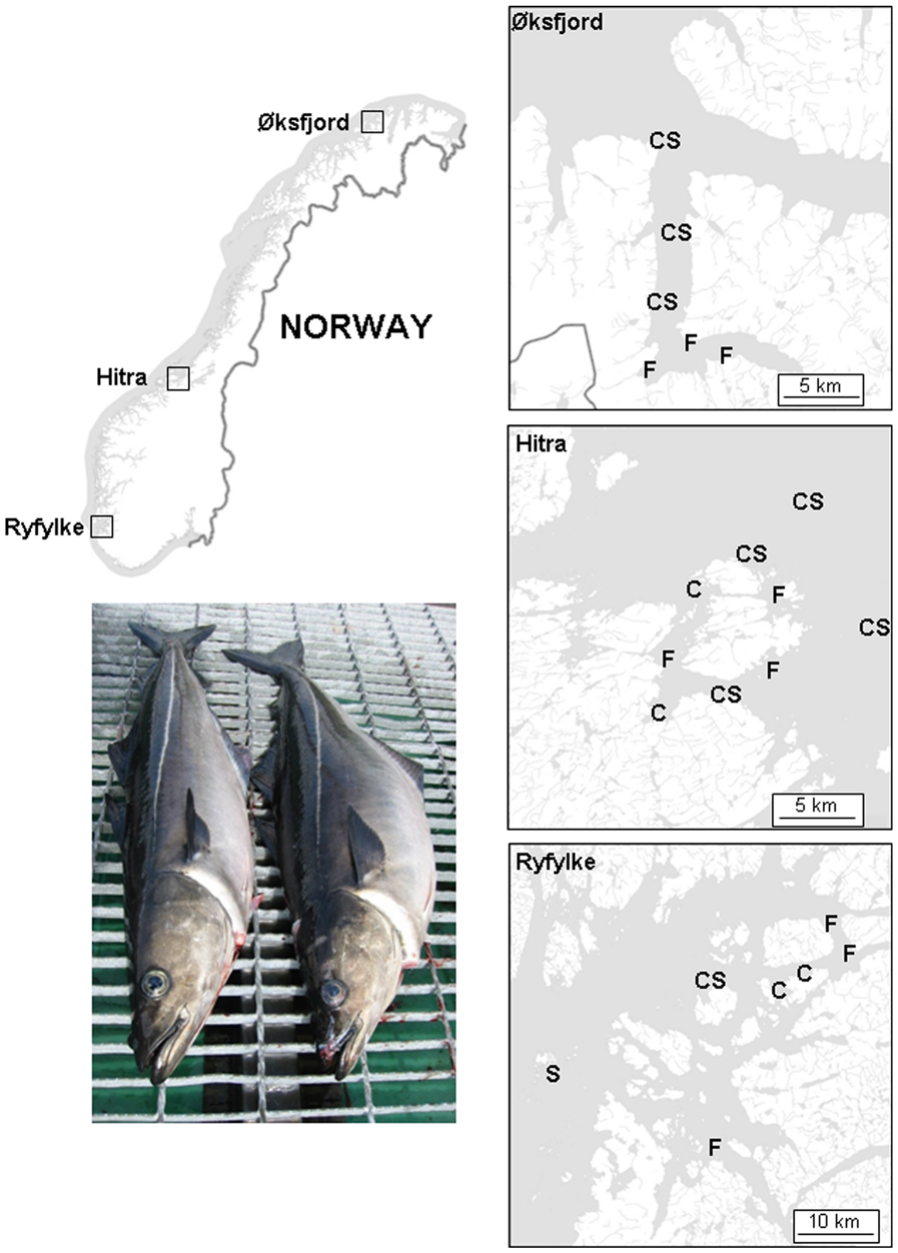
Map of the study locations in the three Norwegian salmon farming areas of Ryfylke, Hitra and Øksfjord. (F) = salmon farm sampling location for both saithe *Pollachius virens* and Atlantic cod *Gadus morhua*; (S) = non-farm sampling location for saithe; (C) = non-farm sampling location for Atlantic cod. The picture shows an un-associated (left) and farm-associated (right) saithe of similar length but distinctly different morphology sampled from Hitra.

Saithe and cod were sampled from the three salmon farming areas (Ryfylke 59°N, Hitra 63°N and Øksfjord 70°N) from the same Atlantic salmon (*Salmo salar*) farms and during the same season (summer) as aggregation sizes were determined [6]. Within each salmon farming area, fish were sampled at three farms and two to six non-farm control locations (Fig. 1). Farm-associated fish were captured within 5 m of cages containing salmon. The number of non-farm locations varied from two to six depending on the area and species of wild fish sampled (Saithe: Ryfylke 2, Hitra 4, Øksfjord 3; Cod: Ryfylke 3, Hitra 6, Øksfjord 3). Control fish were sampled from locations 4 to 20 km distant from the nearest farm (Fig. 1) to limit the possibility of sampling fish at non-farm locations that had interacted recently with a farm. The 4 km minimum limit was based on telemetry-derived observations of the predominant movements of wild cod and wild saithe [9], [10], [11] in the vicinity of fish farms.

All fish were sampled with standardised hook and line fishing gear. Collections by hook and line select for feeding fish, but are more suitable for accurate counts of the number of external parasites than other catch methods such as trawling or gill nets which may remove external parasites through abrasion. Moreover, capture by any other method beside the cages at fish farms is impractical due to possible negative interactions of fishing gear with fish farming structures. Collections were made at each location from June to September 2007 during the period where feed input to salmon farms is high [7].

### Size, diet and condition indices

Upon capture, fish were immediately examined for the presence of external parasites (see parasite sampling section below) and then placed on ice. Fish were weighed and measured to the nearest 0.5 cm (fork length; FL). Each fish was dissected and liver and gonad weights were obtained. Sex for each fish was determined by macroscopic examination of the gonads. In gadoid species, such as Atlantic cod, lipids are stored primarily in the liver [16] making liver weight a measure of spawner quality [17]. Therefore, we calculated three condition indices: body condition, the hepatosomatic index and the gonadosomatic index. Fulton's condition index (FCI) was calculated with the formula: FCI = (W/FL^3^)×100, where W = wet weight–stomach content weight and FL = fork length (cm). The hepatosomatic index (HSI) was calculated using the formula: HSI = (LW/W)×100, where LW = liver weight and W = wet weight–stomach content weight. The gonadosomatic index (GSI) was calculated using the formula: GSI = (GW/W)×100, where GW = gonad weight and W = wet weight–stomach content weight.

Stomach contents from the foregut were examined and prey species were identified to the lowest taxonomic level possible and weighed. Prey categories were later reduced to 11 for saithe (waste salmon feed, Brachyura, Osteichthyes, Polychaeta, Caridea, zooplankton, Phaeophyceae, Bivalvia (principally *Mytilus* sp.), Ophiuridae, Hydroida (principally *Ectopleura larynx*), and other organic matter) and 13 for cod (waste salmon feed, Brachyura, Osteichthyes, Polychaeta, Caridea, Phaeophyceae, Bivalvia (*Mytilus* sp.), Holothuria, Ophiuridae, Echinoidea, Octopoda, Amphipoda and other organic matter).

### Parasite sampling

Fish were examined to estimate the incidence of parasites that may have occurred in increased incidence around fish farms through direct transfer from the farmed salmonids (e.g. mobile sea lice) or through indirect means, such as the modified farm environment increasing the density of con-specific fish or the pool of intermediate hosts available to these parasites, thus increasing their incidence. Immediately upon capture, saithe and cod were examined for the incidence of mobile sea lice (*Caligus* spp.) and attached parasitic copepods (*Clavella* sp.) on all external surfaces, and inside the mouth and gills. In August, 100 mobile sea lice from un-associated (hereafter UA) and farm-associated (hereafter FA) fish were collected in all salmon farming areas to identify the species composition of mobile sea lice. We hypothesised that FA cod and saithe would have elevated levels of *Caligus* compared to UA fish either through direct transfer of adult *Caligus* from caged salmon or elevated levels of *Caligus* larvae in the waters surrounding farms.

Gills of cod were examined for the presence and abundance of *Lernaeocera branchialis*, a copepod parasite of cod which invasively attaches to the gills and feeds on blood [18]. For *Clavella* sp. and *L. branchialis*, we hypothesised that no differences in infestation levels would be detected between FA and UA fish, as no direct transfer route between salmon farms and wild fish has been established for these parasites.

Livers were dissected from both species of fish and inspected for the third stage (L3) larvae of the parasitic nematode *Anisakis simplex* [19]. Infection intensity was scored on a semi-quantitative scale form 0 to 3: 0 = *A. simplex* absent; 1 = mild infestation; 2 = moderate infestation; and 3 = heavy infestation. We hypothesised that L3 larvae of *A. simplex* would be less abundant in FA than UA fish as high consumption of lost feed at farms would mean lower consumption of natural prey items such as crustaceans, squid and fish, which may contain L3 larvae.

### Statistical analyses

As gonadal development was minimal during the non-spawning season sampling period and diets in the non-spawning season are not known to vary among male and female cod and saithe, we pooled the sexes for dietary analyses and analyses of condition. Further, as differences in the incidence of parasites among male and female gadoids have rarely been found [20], and no differences are known for the parasite species investigated here, we pooled the sexes for parasite analyses.

Non-parametric multivariate techniques were used to compare dietary compositions among farm and non-farm locations. All multivariate analyses were performed using the PRIMER statistical package. Prior to calculating the Bray-Curtis similarity matrices, the dietary data were pooled across all individuals sampled within each location and month by summing the total weights of prey items within each prey category to reduce the stress of MDS representation. Fourth root transformations were made to weigh the contributions of common and rare dietary categories in the similarity coefficient. Non-metric multidimensional scaling (nMDS) was used as the ordination method. Variables that had more influence on similarities within groups and dissimilarities among groups of locations or depths, determined by ANOSIM (analysis of similarity), were calculated using the SIMPER (similarity percentages) procedure. The ANOSIM permutation test was used to assess the significance of differences among farm and non-farm locations. As diets of both saithe and cod at farms contained feed pellets, we repeated all analyses with this prey category removed to determine if differences in diet among farm and non-farm locations remained significant.

To test for differences in fish size (fork length; FL), stomach content weight, FCI, HSI, GSI and the incidence of the various parasites among farm and non-farm locations in each of the three fish farming areas, we used Generalized Linear Models (GLMs). Prior to the GLMs, heterogeneity of variance was tested with Cochran's *C*-test. Data were ln(*x*+1) transformed if variances were significantly different at p = 0.05. Comparisons across fish in all size classes were made in each of the three farming areas for cod. To ensure that any differences detected in comparisons were not related to the different sizes of fish in the FA and UA treatments, we used FL as a co-variate in analyses of stomach content weight, condition and parasite loads. For saithe, as HSIs>10% are indicative of a waste feed dominated diet for several months and wild saithe fed solely on natural diets do not have HSIs>10% [21], we tested if the incidence of the various parasites differed among FA fish with HSIs>10%, FA fish with HSIs<10%, and UA fish with HSIs<10%. To detect if the parasite loads we detected were related to the body condition (FCI) of wild fish, we applied multiple regression analysis for both cod and saithe.

## Results

### Size structures of farm-associated and un-associated fish

In total, 355 FA and 215 UN saithe were captured at sizes ranging from 21.5–108.5 cm fork length (FL) and weights from 0.1–12.5 kg. 171 FA and 178 UA cod were collected at sizes ranging from 28.5–121.0 cm FL and weights from 0.23–18.0 kg. Saithe were captured at all farms, while cod were only available at 8 of the 9 farms (all except one farm at Hitra). Significant differences were detected in mean fork lengths among UA and FA groups for both species in all three farming areas (Table 1). FA saithe were larger than UA saithe at two of the three farming areas (Hitra and Øksfjord), but significantly smaller at Ryfylke. FA cod were significantly larger than UA cod in Ryfylke and Hitra but not Øksfjord, and the magnitude of the difference varied greatly among the areas.

**Table 1:** Mean sizes of samples of saithe (*Pollachius virens*) and Atlantic cod (*Gadus morhua*) used to compare diet, condition and parasite loads across farm-associated (FA) and farm unassociated (UA) locations in each of the three Norwegian salmon farming areas.

	FA/UA	Ryfylke		Hitra		Øksfjord	
		n	FL (cm)	n	FL (cm)	n	FL (cm)
***P. virens***	FA	97	50.1±0.7**^b^**	148	40.2±1.2**^a^**	110	46.2±0.8**^a^**
	UA	30	54.2±0.9**^a^**	88	34.3±1.1**^b^**	97	43.4±0.8**^b^**
***G. morhua***	FA	13	63.3±4.5**^a^**	89	52.8±1.6**^a^**	65	62.3±2.5
	UA	12	46.7±4.4**^b^**	75	45.7±1.9**^b^**	91	58.5±1.5

Superscripts (^a,b^) indicate a significant difference was detected between the FA and UA groups at p<0.05.

### Diets of farm-associated and un-associated fish

Saithe captured from non-farm locations had a higher proportion of empty stomachs (31%) and lower average stomach content weight (8.6 g) compared to FA saithe (16%, 20.2 g). For cod, both FA (18%) and UA fish (19%) had similar proportions of empty stomachs, although stomach content weight was higher in FA (32.9 g) than UA fish (23.2 g). 44.3% of saithe and 20% of cod captured around farms had waste feed in their stomachs. Overall, waste feed accounted for 71% (14.2 g) and 25% (8.3 g) of the diet by weight of FA saithe and cod, respectively.

**Figure 2:**
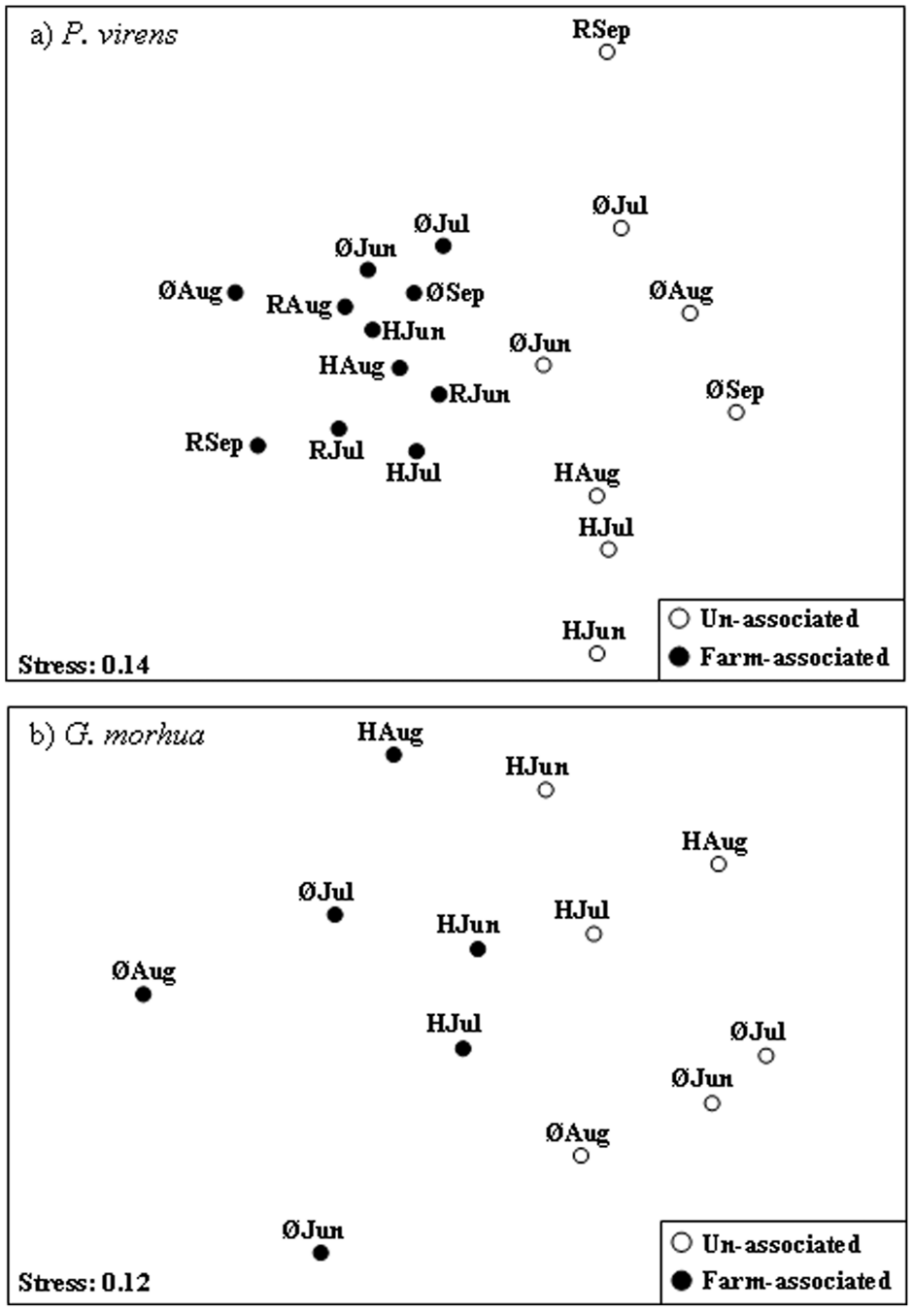
Non-metric multi-dimensional scaling plots of dietary items of saithe *Pollachius virens* and Atlantic cod *Gadus morhua* sampled from farm and non-farm locations throughout Norway from June to September. a: saithe; b: Atlantic cod. Each point is based on mean weights of prey categories for the specific month. Jun = June; Jul = July; Aug = August; Sep = September. R = Ryfylke; H = Hitra; Ø = Øksfjord.

The 2-dimensional nMDS plot based on weights of prey groups by location and month revealed clear separation of the diets of FA and UA fish for both saithe (Fig. 2a) and cod (Fig. 2b). ANOSIM indicated that differences in diets between FA and UA fish were significant (saithe: *R*_global_ = 0.69, p = 0.001; cod: *R*_global_ = 0.45, p = 0.003). When pellets were removed from the analysis, differences in diets between FA and UA fish remained significant (saithe: *R*_global_ = 0.52, p = 0.01; cod: *R*_global_ = 0.38, p = 0.02).

Diets of FA saithe clustered together, regardless of sampling location and month, while diets of UA saithe were more variable (Fig. 2a). UA saithe diets were characterised by similar weights of relatively few dietary items. Over 70% of group similarity was accounted for by fish (41.5%), zooplankton (16.8%), crustaceans (8.0%) and ophiuroids (4.5%). Over 80% of similarity in FA saithe diets was due to waste feed (45.7%), fish (14.8%), mussels (10.5%) and zooplankton (10.2%). Dissimilarities in diets between UA and FA saithe were due to large differences in the abundance of a few of the major items (waste feed 32.3% F>C, fish 14.3% C>F, zooplankton 10.3% F>C and mussels 9.9% F>C).

Similarities in UA cod diets were predominantly due to similar weights of fish (39.7%), crabs (24.3%), ophiuroids (9.7%) and crustaceans (6.5%) while similarities in FA cod diets were predominantly due to fish (37.6%), polychaetes (19.6%), pellets (14.6%) and crabs (9.6%). FA cod consumed more waste feed, polychaetes and fish (dissimilarities of 18.9%, 12.1% and 7.8%, respectively) while UA cod consumed more Ophiuridae, crabs and mussels (dissimilarities of 11.5%, 9.9% and 7.8%, respectively).

### Body, liver and gonad condition of farm-associated and un-associated fish

**Figure 3:**
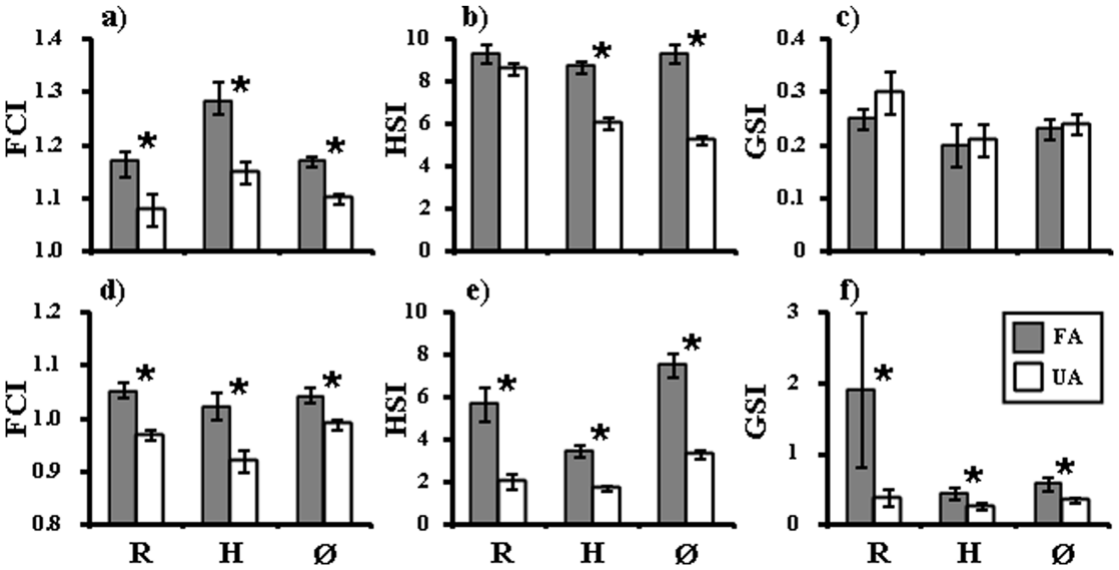
Condition indices of farm-associated (FA) and un-associated (UA) saithe *Pollachius virens* and Atlantic cod *Gadus morhua* in each of the three intensive fish farming areas. a, b, c: saithe; d, e, f: Atlantic cod. R = Ryfylke; H = Hitra; Ø = Øksfjord. FCI = Fulton's condition index; HSI = Hepatosomatic index; GSI = Gonadosomatic index. * indicates a significant difference at p<0.05 was detected among the groups.

FA saithe had significantly higher average FCIs (1.06-1-12 times) than UA fish in all three farming areas (Fig. 1, Fig. 3a). Average HSIs were significantly higher in saithe (1.4–1.8 times) collected around farms compared to UA fish at Hitra and Øksfjord (Fig. 3b). No difference was detected for FA and UA fish sampled from Ryfylke. As the June-September sampling period occurred after the main spawning period for saithe and many of the individuals sampled were less than 2 kg in size and thus likely to be immature, no difference was detected in average GSIs among farm and UA fish in any of the three areas (Fig. 3c).

FCIs, HSIs and GSIs of cod were clearly affected by association with salmon farms. FCIs were consistently 1.06–1.11 times greater in FA than UA cod in all three areas (Fig. 3d). Similarly, average HSIs varied among the three areas, but were consistently 2.0–2.8 times greater in cod collected around farms compared to UA fish (Fig. 3e). In contrast to saithe, where average GSIs in FA and UA fish were similar, average GSIs in cod were significantly greater (1.7–4.8 times) in FA than UA cod in all three areas, despite the timing of sampling in the post-spawning period (Fig. 3f).

### Parasite loads of farm-associated and un-associated fish

**Figure 4:**
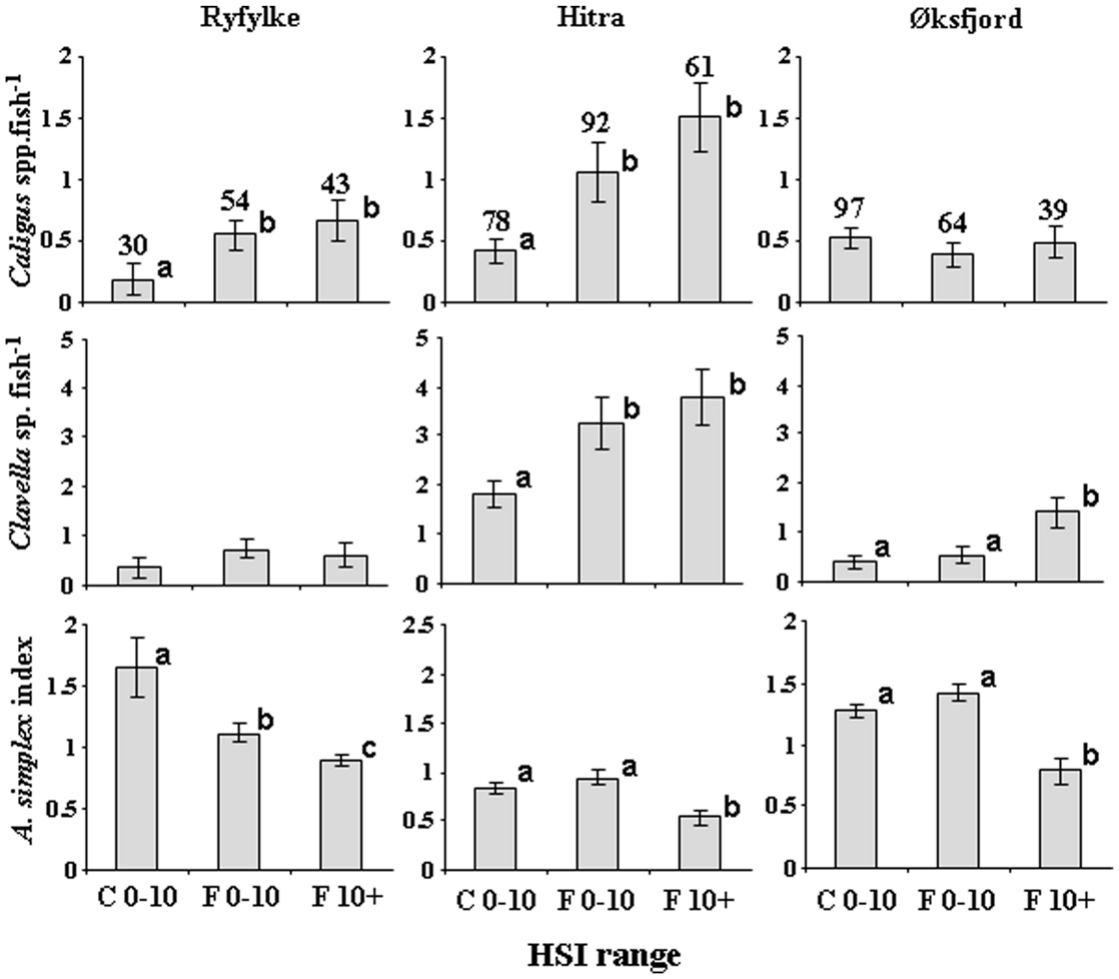
Mean abundances (± SE) of common parasites of saithe *Pollachius virens* in fish with a hepatosomatic index (HSI)<10 taken from non-farm locations (UA), and fish with HSI<10 and HSI>10 captured in association with Atlantic salmon farms (FA) in the three intensive fish farming areas. HSI = Hepatosomatic index. Superscripts (^a,b,c^) indicate a significant difference was detected among the groups at the p<0.05 level. Numbers above bars give the number of fish sampled for each comparison.

Significant differences in the abundances of parasites were detected in both directions, with FA or UA fish having greater levels of particular parasites in certain fish farming areas. From the collections in August, two species of mobile sea-lice were identified on both saithe and cod in all three areas: *Caligus elongatus* and *C. curtus*. Significantly higher numbers of sea lice occurred on FA saithe with HSIs>10 or <10 compared to UA fish with HSIs<10 at Ryfylke (2.5 to 3.5 times) and Hitra (3.1 to 3.7 times), but not at Øksfjord (Fig. 4). *Clavella* sp. abundances were significantly higher in FA saithe with HSIs>10 or <10 compared to UA fish at Hitra (1.8 to 2.1 times). FA saithe with HSIs>10 had 2.6 to 3.6 greater abundances of *Clavella* sp. than both FA saithe with HSIs<10 and UA fish in Øksfjord. No differences in *Clavella* sp. abundance among the three groups were detected at Ryfylke. For the *Anisakis simplex* index, FA saithe had consistently lower values that UA saithe across the three locations. FA saithe with HSIs>10 had 1.6 to 2.1 times lower *A. simplex* infestations than FA saithe with HSIs<10 and UA fish.

**Figure 5:**
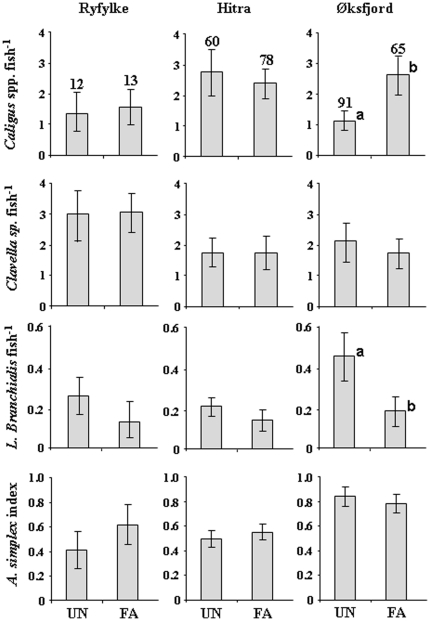
Mean abundances (± SE) of common parasites of farm-associated (FA) and un-associated (UA) Atlantic cod *Gadus morhua* in each of the three intensive salmon farming areas. HSI = Hepatosomatic index. Superscripts (^a,b^) indicate a significant difference was detected between the two groups at the p<0.05 level. Numbers above bars give the number of fish sampled for each comparison.

*Caligus* spp. occurred in abundances 2.4 times higher on FA cod at Øksfjord compared to UA cod, whereas no significant differences between FA and UA cod were detected at Ryfylke and Hitra (Fig. 5). No significant differences were detected for *Clavella* sp. or *Anisakis simplex* L3 larvae between farm-associated and UA fish in any of the three areas. The gill parasite *Lernaeocera branchialis* occurred in significantly higher abundance (2.8 times) in UA cod than FA cod in Øksfjord, with no difference detected in the other two areas.

Multiple regression analysis of parasite loads versus body condition revealed that none on the four species of parasites investigated for cod were significantly related to FCI (F = 1.12, p = 0.35; R^2^ = 0.02; *Caligus* spp.: p = 0.11; *Clavella* sp.: p = 0.17; *L. branchialis*: p = 0.86; A. *simplex*: p = 0.49). For saithe, the multiple regression was significant (F = 9.7, p<0.001; R^2^ = 0.05), with *Clavella* spp. positively related to FCI (p = 0.003), *Anisakis* sp. strongly negatively related to FCI (p<0.001), and no relationship evident for mobile sea lice (*Caligus* spp.: p = 0.59).

## Discussion

### Proxy measures of fitness of farm-associated and un-associated fish

We have demonstrated that proxy measures of fitness (FCI, HSI, abundances of specific parasites and diet) of wild saithe and cod caught in close association with salmon farms differ significantly from their counterparts captured distant from farms. These effects are likely to be general across the spatial extent of salmon farming in Norway (59°N–70N°) and apply to a substantial pool of fish aggregated around farms. Dempster et al. [6] conservatively estimated that over 12000 tons of wild fish, principally saithe and cod, were aggregated at Norway's 1198 salmon farms on any given day in summer based on video-derived estimates of aggregations at the same 9 farms investigated here. Conclusions derived from this study are therefore based upon these abundance estimates, but are limited to the summer months during which samples were taken.

Salmon farms clearly increased the amount of food consumed by closely associated saithe and cod, indicating a strong trophic link between farms and wild fish. Stomachs of FA saithe contained more than twice the amount of food by weight than UA fish with stomach content weight similarly elevated in FA cod (1.4 times). Food pellets are high in fish proteins and oils and thus provide a high energy source of feed [22], although with distinctly different fatty acid distributions from natural diets [13]. While waste feed dominated diets of FA saithe and cod, the composition of dietary items still differed among FA and UA fish when waste pellets were removed from analyses, indicating that the availability of other types of prey differed between farm and non-farm locations. Salmon farms are known to have modified meio- and macro-fauna communities [23] and modified fish assemblages [6] compared to control locations, which likely contributed to the dietary differences.

The increased body and liver condition observed in FA saithe and cod is likely linked to the trophic subsidy that farms provide. Livers are the principal lipid and thus energy stores in gadoids [16]. High HSIs are indicative of high total lipid energy, which is known as a direct proxy to egg production in gadoid fish [17]. Moreover, lipid energy reserves 3–4 months prior to spawning are the best proxy for fecundity [24]. In this context, association with fish farms throughout summer and autumn could increase the fecundity of saithe and cod, which spawn in early spring, even if these fish migrate away from farms months prior to spawning.

While fecundity, in terms of egg numbers or size, may increase through FA fish having high energy reserves, the composition of stored lipids in FA saithe and cod may differ from those of UA fish which consume a natural diet (Fernandez-Jover et al. unpubl. data). This may effect egg quality as farm-feeds contain low proportions of highly unsaturated fatty acids (HUFAs) and arachidonic acids, which are key to fertilization rates and egg quality [25]. If the waste-feed dominated diet alters the fatty acid composition of saithe and cod livers and has a negative effect upon egg quality during vitellogenesis, the increased condition evident in FA fish may not translate to a proportional increase in spawning success. Experimental manipulations of wild saithe and cod fed diets containing different proportions of waste feed for various durations and the subsequent evaluation of the effect this has on egg and larval quality are required to determine the extent of this potentially negative effect.

Some parasites were found in elevated abundances in FA fish. We hypothesised that mobile sea-lice would occur in higher abundances on FA fish due to direct transfer or greater infestation levels as larvae occurred in greater abundance. This was the case for saithe at Hitra and Ryfylke and cod in Øksfjord. Similarly the attached copepod *Clavella* sp. was detected in elevated abundances in FA saithe at Hitra and Øksfjord. In contrast to the mobile sea-lice and *Clavella* sp. loads, the gill parasite *Lernaeocera branchialis* and the internal parasite *Anisakis* simplex were only ever detected in lower levels in FA fish. For *L. branchialis*, significant differences between FA and UA fish were only detected in Øksfjord, where UA cod had higher levels. Significantly lower *A. simplex* infections occurred in FA saithe with HSIs>10 in all three farming areas, suggesting that the longer-term residence at salmon farms required to generate an HSI>10 [21] plays an important role in reducing the level of *A. simplex* infection. The strong trophic link between saithe and fish farms, with saithe diets containing >70% by weight of lost feed pellets which are free of *A. simplex* A3 larvae, reduces the amount of potential hosts of A3 larvae such as small fish and crustaceans that saithe consume [19].

Elevated levels of *Caligus* spp. and *Clavella* sp. detected in FA fish may have had detrimental effects upon condition. Limited information exists to assess the threshold levels at which *Caligus* spp. and *Clavella* sp. infestations cause reductions in condition in cod and saithe, although heavy infestation of *Clavella adunca* can produce a moderate reduction in cod condition [20]. However, mobile sea-lice infestations of gadoids were generally close to the range of those typically recorded in Norwegian fjord and coastal waters (1 to 2 *C. elongatus* gadoid^−1^; [26]). *L. branchialis* is considered the most serious metazoan parasite of wild cod [18], [20] and can cause mortality, loss of condition and affect reproductive output. Similarly, heavy *Anisakis simplex* infestation has the capacity to reduce the condition of wild gadoids [20]. The reduction of both of these parasites in FA fish at some locations was therefore likely to have led to increased average condition compared to control fish. However, multiple regression analyses revealed that farm-modified parasite loads did not have major effects on the somatic condition of cod. For saithe, *Clavella* sp. abundance was positively correlated with condition, while the *A. simplex* infestation index was strongly negatively correlated with condition. Regardless of these relationships, body condition was significantly higher for FA fish than UA fish for both cod and saithe across all farming locations. As the body condition index integrates all factors that influence the condition of a fish over its recent life history, including the effects of parasites upon condition, our data suggests that the trophic subsidy that farms provide elevates body condition such that any effects on condition related to modified parasite loads were negligible in comparison.

In addition to the parasite species investigated here, gadoid fish such as *Gadus morhua* and *Pollachius virens* are infected by over 100 pathogens and parasites, at least 20 of which may be directly transferred among salmonids and gadoids [27]. These include some of the most significant diseases prevalent in salmon aquaculture, including *Vibrio anguillarum*, salmonid alphavirus and infectious pancreatic necrosis virus [28], [29]. If these pathogens are enhanced in wild fish aggregated at fish farms they could negatively affect condition and survival; further research is required in this field.

### Fish farms: ecological traps or population sources for wild fish?

In contrast to the detrimental effects of salmon farming detected at the population-level for wild salmonids (sea lice: [30], [31], [32]; escapes: [33], [34]), we did not detect significant negative effects of the co-occurrence of wild saithe and cod with salmon farms. The diet and condition data indicate that wild saithe and cod benefited from their associations with salmon farms through access to greater amounts of food which translated to enhanced condition. While *Caligus* spp. and *Clavella* sp. loads were elevated at some farming locations compared to controls and *Anisakis* sp. and *Lernaeocera branchialis* loads were lowered at some farming locations compared to controls, it appears that any effects these modified parasite loads may have had on the condition of wild cod and saithe were overridden by the trophic subsidy that farms supply. The results provide no evidence that salmon farms act as ecological traps for wild cod and saithe that aggregate in their vicinity, provided that: 1) the modified fatty acid distributions and elevated organohalogen levels in fat stores in livers that results from a fish farm modified diet [35], [36] does not negatively affect physiological processes, vitellogenesis or egg and larval quality; 2) salmon farms do not amplify any of the numerous pathogens not investigated here that salmonids and gadoids share [27]; and 3) that attraction to farms does not disrupt natural spawning migrations or behavior. Future research should seek to discern the effects of both salmon and cod farms during the spawning season for cod resident in fjords containing farms, as a range of different effects are possible during this period, including mass spawning of farmed cod in cod farms [37] and possible avoidance of fjords containing salmon farms by spawning cod [38].

As saithe and cod condition is enhanced by farms, an opportunity exists to protect wild fish around salmon farms where they are aggregated and vulnerable to fishing, thus enabling farms to act as a population source. Presently, fishing adjacent to salmon farms occurs [39], although the importance of this activity to overall catches is unknown. Stocks of fjord cod in southern Norwegian waters, in particular, are depressed due to chronic overfishing [40]. Therefore, to ensure farms do not act as ecological traps for cod via increased fishing mortality alone, restrictions on the fishing of cod in the vicinity of farms could be introduced. Spatial protection from fishing would allow an opportunity for the enhanced condition that cod and saithe generate due to their association with salmon farms to translate to enhanced spawning success. Fish farms have recently become targets of significant fishing pressure [41] in other coastal ecosystems, thus the principle of restricting fishing around farms to ensure they do not function as ecological traps may be broadly applicable. As large, multi-specific aggregations of wild fish aggregate around coastal fish farms wherever they occur [5], [6], we predict that significant conservation benefit would be derived through the protection of tens of thousands of tons of wild coastal fish in high spawning condition if this measure were implemented worldwide.
